# Mask-wearing behavior during COVID-19 in China and its correlation with e-health literacy

**DOI:** 10.3389/fpubh.2022.930653

**Published:** 2022-07-22

**Authors:** Wei Zhang, Shu-Fan Chen, Kun-Kun Li, Huan Liu, Hai-Chen Shen, Xian-Cui Zhang

**Affiliations:** ^1^Department of Neurosurgery Intensive Care Unit, The First Affiliated Hospital of Wannan Medical College, Wuhu, China; ^2^Department of Nursing, Soochow University, Suzhou, China; ^3^Department of Critical Care Medicine, The First Affiliated Hospital of Wannan Medical College, Wuhu, China; ^4^Department of Blood Purification Center, The First Affiliated Hospital of Wannan Medical College, Wuhu, China; ^5^Department of Urology, The First Affiliated Hospital of Wannan Medical College, Wuhu, China; ^6^Department of Health Management Center, The First Affiliated Hospital of Wannan Medical College, Wuhu, China

**Keywords:** COVID-19, public, mask, behavior, e-health literacy

## Abstract

**Background:**

During the Coronavirus **(**COVID-19) pandemic, wearing masks became crucial for preventing infection risk and maintaining basic health. Therefore, it is necessary to understand the behavioral characteristics of the mask-wearing public to provide theoretical reference for the prevention and control of COVID-19.

**Methods:**

We conducted a nationwide survey on the mask-wearing behavior of the public and their health literacy levels by distributing electronic questionnaires. Univariate and logistic regression analyses were performed to determine the factors influencing mask-wearing behavior. Pearson's correlation was used to analyze the correlation between mask-wearing behaviors and e-health literacy.

**Results:**

A total of 1,972 valid questionnaires were collected; 99.8% of the public wore masks when going out and 59.2% showed good mask-wearing behavior. Most people choose to wear disposable medical masks (61.3%), followed by medical surgical masks (52.9%). All participants indicated that they had understood the information on how to use masks, and most obtained it through social media (61.8%). The average of the e-health literacy scores of those with good mask-wearing behavior was significantly higher than those with poor mask-wearing behavior (*P* < 0.05), and each item score of the former's e-health literacy was significantly higher than the latter (*P* < 0.05). Further, there was a positive correlation between mask-wearing behavior and e-health literacy (*R* = 0.550, *P* < 0.05). Logistic regression analysis showed that seven factors are related to mask-wearing behavior, including gender, place of residence, educational level, work or living environment, marital status, flu symptoms, and whether living with people in home quarantine (*P* < 0.01).

**Conclusion:**

The overall compliance of the public's mask-wearing behavior in China during COVID-19 is good. However, there are shortcomings regarding the selection, use, and precautions. The differences in mask-wearing behavior are related to factors including gender, place of residence, educational level, work or living environment, marital status, presence of flu symptoms, and whether living with people in home quarantine. Higher levels of e-health literacy indicated better mask-wearing behavior. Therefore, it is necessary to strengthen the public's popularization and education regarding the prevention and control of COVID-19.

## Introduction

The novel coronavirus disease (COVID-19), the third coronavirus pandemic, can cause symptoms such as fever and pneumonia. With the development of the pandemic and the deepening of related research, the World Health Organization (WHO) identified the causal virus as the severe acute respiratory syndrome coronavirus 2 (SARS-CoV-2) and declared COVID-19 as a public health emergency of international concern (1). The first case of COVID-19 appeared in Wuhan on December 8, 2019, and then the epidemic broke out in China. Through 22 April 2022, it was reported that there were 199,074 confirmed COVID-19 cases in China (2). Regarding the current knowledge, the source of infection is patients and asymptomatic infections; the main transmission is *via* respiratory droplets followed by contact transmission.

At the same time, other transmission routes such as aerosol and digestive tract caused by feces and urine should be paid attention (3). Experts pointed out that if personal protection and disinfection are well-addressed, COVID-19 is preventable and controllable, and recommended wearing masks in public environments as an important measure to prevent and control epidemic diseases transmitted by the respiratory tract (4). Siegel et al. (5) showed that physical block is effective in preventing the transmission of droplets over short distances, while preventive measures of more complexity are required to prevent smaller airborne particles. As a physical way to block virus infection, masks can block the spread of pathogens through droplets, preventing the transmission of pathogens from virus carriers to patients and reducing infection risk caused by their inhalation, and has a two-way isolation protection effect (6). Davies et al. (7) showed that masks could prevent the work environment's contamination during outbreaks of influenza or other droplet-borne infectious diseases by reducing aerosol transmission and the risk of wearer's exposure to body fluids (including blood, secretions, and excrement) through the nose and mouth. MacIntyre et al. (8) showed that increased use of facemasks during the influenza pandemic significantly reduced spread in households. Therefore, wearing masks is crucial to prevent the risk of COVID-19 infection and maintain basic health.

With the popularization of information technology, people mostly obtain health-related information through the Internet currently. Wang et al. (9) showed that the electronic health (e-health) literacy level directly affects the residents' ability to obtain health-related information through the Internet. E-health literacy refers to the ability to obtain, understand, and evaluate health information from electronic resources, and use it to process and solve health problems, including a series of technology-based health tools for effective use of vital skills and knowledge (10). During COVID-19, community residents mainly obtained disease-related knowledge through instant messaging, news apps, government department websites, radio, TV news, and other channels (11, 12), and e-health literacy level plays an important role in identifying true and false information about COVID-19 epidemic (13). Notably, improving the public's e-health literacy level will be beneficial for individuals to acquire health knowledge purposefully, and effectively apply for the self-protection, disease prevention, and health check-ups (14), so as to improve and enhance individual health behaviors.

The correct selection and use of suitable respiratory protective equipment is a key for people to protect their health effectively and reduce the spread of infectious diseases. The Chinese government recommended wearing masks at the beginning of the pandemic and launched a series of policies to guide the public wear masks scientifically. Currently, the types of masks mainly include medical protective, particulate protective, medical surgical, disposable medical, and ordinary masks. Due to differences in their materials and production standards, the public should choose appropriate masks based on different exposure scenarios (15). However, the behavioral pattern of people wearing masks and COVID-19's impact on it are unclear. Experts warned that the lack of knowledge about the correct use of masks might lead to irrational selection and use, which could contribute to waste of resources and increase the infection risk (16, 17). Therefore, this study investigated and analyzed the mask-wearing behavior of the public during COVID-19 to understand its relationship with health literacy through a survey of the national population. It also aims to provide basic information for the precise prevention and control of COVID-19, and the theoretical support for the mask-wearing behavior during COVID-19.

## Methods

### Study design

**Figure 1:**
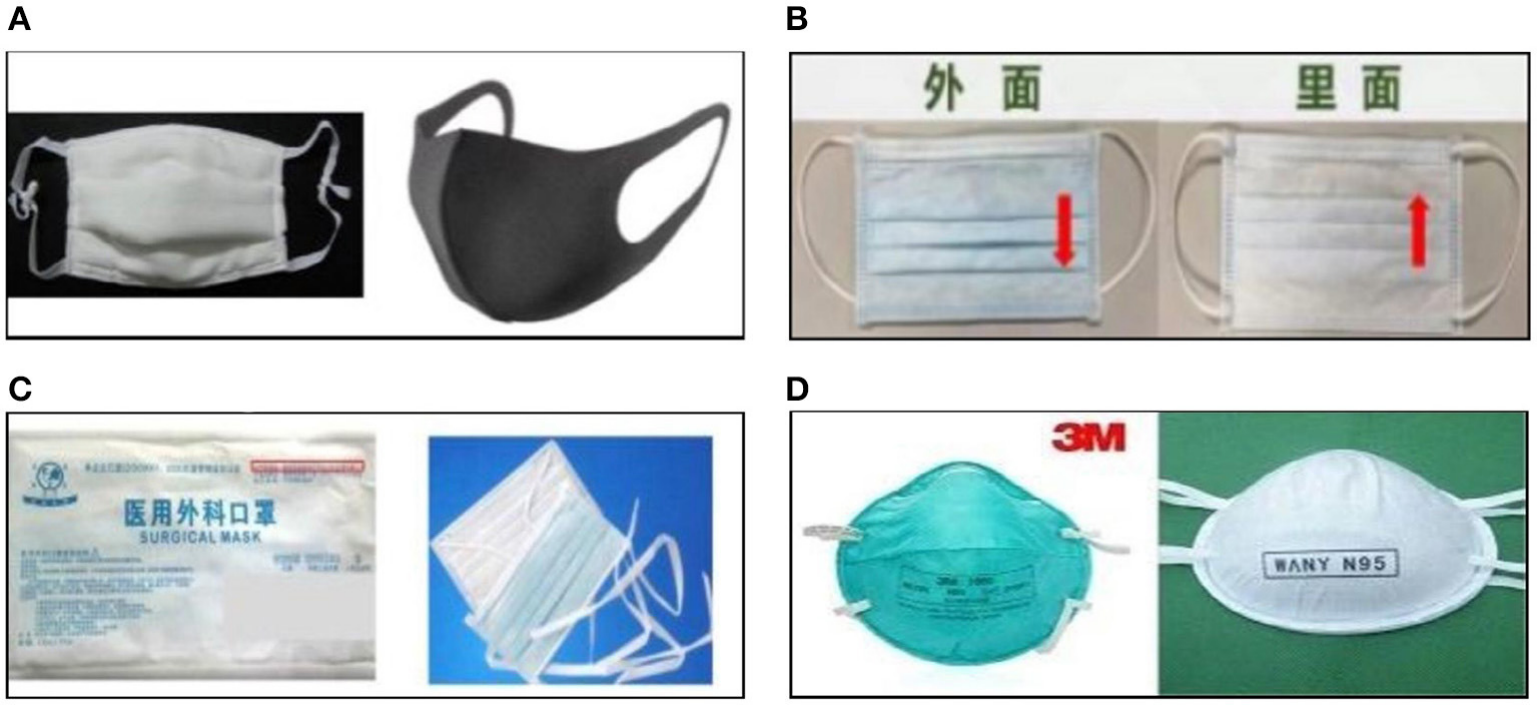
The masks types used in this study. **(A)** Ordinary mask. **(B)** Disposable medical masks. **(C)** Medical surgical masks. **(D)** Medical protective mask.

This study is a cross-sectional survey that was conducted nationwide in China from November to December 2021. It adopted the convenience sampling method to select the public who live in China and understand Chinese as the study subject, while medical and health care personnel were excluded. Firstly, we developed electronic questionnaires through an online crowdsourcing platform, Wenjuanxing, in mainland China that provides functions equivalent to Amazon Mechanical Turk. Secondly, we released the recruitment notice of investigators for this study and applicants should be non-healthcare occupations, with a bachelor degree or above, be interested in scientific research and have certain scientific research experience, and have sufficient time to complete this survey. Finally, we recruited 15 investigators. After uniform training of investigators, the scope of the survey was diffused outward through their social connections and was circulated in their social networks. We set up the permission to fill in the questionnaire, each IP address can only be allowed to fill in and submit once to ensure no duplication of the survey. To reduce the survey bias, the mask type was represented by pictures in the questionnaire (Figure 1).

In this survey, we distributed questionnaires through WeChat and QQ, and the purpose and significance were introduced first to obtain the respondents' cooperation. Then they were informed of the method of filling out the questionnaire, which completed the survey. The time to fill in the questionnaire was ~10 min, and the participant could submit it only after completely filling it. For interruptions or repeat attempts, the questionnaire could not be submitted successfully. With the support of the researcher's own project fund, after submitting the questionnaire, the participant could participate in the lottery, and randomly get cash, electronic coupons and other prizes in return.

### Instrument

Two questionnaires were used in this study, the “Questionnaire on Face Masks Use for the Public (Except Healthcare Workers)” and the “e-Health Literacy Scale (e-HEALS)” which mainly surveyed the public's mask-wearing behavior and their health literacy during COVID-19 in China.

The “Questionnaire on Face Masks Use for the Public (Except Healthcare Workers)” was compiled by the West China Hospital, Sichuan University (18). It was developed according to the guidelines for the public to wear masks by the National Health Commission of China (19) and advice of wearing a mask for the public in the context of COVID-19 by WHO (20). It comprised two parts: (a) general information, with eight items, including gender, age, place of residence, education level, work or living environment, marital status, presence of flu symptoms such as coughing or sneezing, and whether living with a COVID-19 patient who has been discharged from the hospital and has been in home quarantine in the past week; (b) mask-wearing behaviors, with 20 items, including single-choice and multiple-choice questions. The specific calculation method is as follows: Single-choice questions used a 4-point Likert scale, wherein “1” means “never,” “2” means “occasionally,” “3” means “often” and “4” means “every time.” Among them, a total of 7 items with positive scoring, including items 5, 6, 7, 8, 10, 12 and 15, responses of “never” and “occasionally” were defined as “poor,” while “often” and “every time” were defined as “good;” a total of 6 items with the opposite scoring, including items 9, 11, 13, 14, 16, and 17. Besides, items 10 and 12 are sub-items of items 9 and 11, respectively. If the participant chooses “never” for these two items, the two sub-items will be skipped and no points will be scored. For ease of analysis, multiple-choice questions, including items 1, 2, 3, 4, 18, 19, and 20 were not calculated, which are “when to wear a mask,” “what type of masks are selected,” “whether have learned information about how to use masks,” “the sources of information about wearing masks,” “the way to remove masks,” “methods for disposal” and “frequency of changing the mask.” The highest score for the part B was acquired only when participants choose “occasionally” for items 9 and 11, and choose “every time” for items 10 and 12. Therefore, the total score for ranges from 13–50 points. We calculated the minimum score for “good” mask-wearing behavior is 35 points, which means participants choose “every time” in the positive scoring items and “never” in the opposite scoring items, besides, they choose “never” in items 9 and 11. Depending on whether the final score reached 35 or more points, mask-wearing behavior was defined as “good” or “poor,” respectively. We recruited 30 participants to conduct a small sample survey and then conducted a reliability analysis of the questionnaire. The results showed that the overall Cronbach's α co-efficient of the questionnaire was 0.687, indicating that the internal consistency of the scale is acceptable.

The e-HEALS was first compiled by Norman et al. (10), and Guo et al. (21), translated into Chinese, and revised according to Chinese cultural background and language. The Chinese version includes eight items, and each adopts the 5-point Likert scoring method. “1” means “strongly disagree,” and “5” means “strongly agree” and the higher the score, the higher the self-perceived e-health literacy. The Chinese version is divided into three dimensions, including the application ability test of network health information and service, evaluation ability test, and decision-making ability test, and the Cronbach's α co-efficient of the scale was 0.913.

### Statistical analysis

Data were analyzed using SPSS Statistics version 22.0 software (IBM, Armonk, New York, USA). Descriptive statistics were used to summarize demographic data (i.e., means, percentages, standard deviations), and the chi-square test was used to analyze the differences between groups. The significance of each variable was assessed by univariate analysis. The variables with *P* < 0.05 were included in the logistic regression analysis to analyze the influencing factors of mask wearing behavior. Correlation analysis of the public mask-wearing behaviors and the e-health literacy used Pearson's correlation. *P* < 0.05 were regarded as statistically significant.

## Results

Approximately 2,200 questionnaires were distributed (the exact number not counted), and a total of 2,093 questionnaires were collected and the recovery rate was 95.1%. The questionnaires were reviewed by two researchers, and 121 responses with incomplete answers, logic, and data format errors were excluded. A total of 1,972 valid questionnaires were analyzed. As shown in Table 1, the population distribution of participants was divided by different areas, including Northeast China (5.0%), North China (7.4%), East China (51.7%), South China (7.5%), Central China (13.6%), Southwest China (7.9%), and Northwest China (7.0%). Most of participants were female (62.5%) and the participants' mean age was (33.66 ± 9.58) years, 74.4 % of which were young and middle-aged. More than half of participants live in cities (55.2%), with a bachelor's degree (56.0%) and were married (60.5%), and there were no statistically significant differences in the demographic characteristics of participants in different areas (*P* > 0.05).

**Table 1:** Demographic characteristics of participants in different areas.

**Characteristic**	**Category**	**Case [*****n*** **(%)]**	**National population-level (%)**	**Areas [*****n*** **(%)]**							**χ^2^**	***P*** **-value**
				**Northeast China**	**North China**	**East China**	**South China**	**Central China**	**Southwest China**	**Northwest China**		
Gender	Male	740 (37.5)	51.24	44 (44.4)	44 (30.3)	394 (38.7)	45 (30.4)	100 (37.3)	61 (39.1)	52 (38.0)	9.157	0.165
	Female	1,232 (62.5)	48.76	55 (55.6)	101 (69.7)	625 (61.3)	103 (69.6)	168 (62.7)	95 (60.9)	85 (62.0)		
Age	<40	1,468 (74.4)	50.79	68 (68.7)	103 (71.0)	753 (73.9)	111 (75.0)	202 (75.4)	102 (65.4)	95 (69.3)	8.087	0.232
	≥40	485 (24.6)	49.21	31 (31.3)	42 (29.0)	266 (26.1)	37 (25.0)	66 (24.6)	54 (34.6)	42 (30.7)		
Place of residence	City	1,088 (55.2)	40.74	51 (51.5)	68 (46.9)	575 (56.4)	85 (57.4)	149 (55.6)	86 (55.1)	74 (54.0)	7.328	0.835
	Township	462 (23.4)	23.01	22 (22.2)	41 (28.3)	231 (22.7)	31 (20.9)	66 (24.6)	38 (24.4)	33 (24.1)		
	Countryside	422 (21.4)	36.11	26 (26.3)	36 (24.8)	213 (20.9)	32 (21.6)	53 (19.8)	32 (20.5)	30 (21.9)		
Educational level	Primary school or below	82 (4.2)	30.32	5 (5.1)	7 (4.8)	37 (3.6)	5 (3.4)	13 (4.9)	7 (4.5)	8 (5.8)	18.969	0.754
	Junior high school	214 (10.9)	37.03	15 (15.2)	22 (15.2)	105 (10.3)	14 (9.5)	25 (9.3)	20 (12.8)	13 (9.5)		
	High school	232 (11.8)	16.13	13 (13.1)	18 (12.4)	114 (11.2)	12 (8.1)	32 (11.9)	20 (12.8)	23 (16.8)		
	Undergraduate	1,105 (56.0)	7.16	51 (51.5)	77 (53.1)	579 (56.8)	94 (63.5)	152 (56.7)	84 (53.8)	68 (49.6)		
	Post-graduate (Master/Ph.D.)	339 (17.2)	0.82	15 (15.2)	21 (14.5)	184 (18.1)	23 (15.5)	46 (17.2)	25 (16.0)	25 (18.2)		
Marital status	Unmarried	634 (31.7)	21.60	33 (33.3)	43 (29.7)	350 (34.3)	43 (29.1)	80 (29.9)	39 (25.0)	46 (33.6)	14.464	0.272
	Married	1,186 (60.5)	71.33	59 (59.6)	87 (60.0)	589 (57.8)	100 (67.6)	167 (62.3)	105 (67.3)	79 (57.7)		
	Divorced	152 (7.7)	5.69	7 (7.1)	15 (10.3)	80 (7.9)	5 (3.4)	21 (7.8)	12 (7.7)	12 (8.8)		
Total		1,972	–	99 (5.02)	145 (7.35)	1,019 (51.67)	148 (7.51)	268 (13.59)	156 (7.91)	137 (6.95)		
National population-level [*n*× 10^9^ (%)]				0.17 (11.98)	0.10 (6.99)	0.38 (29.98)	0.19 (13.19)	0.27 (15.84)	0.21 (14.53)	0.10 (7.33)		

*There are fewer cases of <18 years old and >65 years old, so the age is divided into two groups for statistical analysis*.

In this survey, almost everyone wore masks, of which 19.0% wore masks at home, 41.6% wore masks outdoors where there is good ventilation and no crowd, 63.1% wore masks when in close contact with people, 74.3% wore masks in densely populated places, and 0.20% did not wear masks. Moreover, all participants have learned information about how to use masks, and the information sources included newspapers (26.8%), TV news (56.4%), radio (36.3%), Internet (47.2%), social media (WeChat, Weibo, QQ) (61.8%), community publicity (26.1%), relatives and friends (20.4%), other channels (15.2%). The number of those who wore disposable medical masks is the highest, and those who wore ordinary masks lowest. Regarding different types of masks that the Chinese public wore during COVID-19 included disposable medical (61.3%), medical surgical (52.9%), medical protective (26.5%), and ordinary (24.7%). The univariate analysis showed that there was a statistically significant difference in the mask-wearing scores between different gender, age, place of residence, educational level, marital status, work or living environment, presence of flu symptoms and whether living with people in home quarantine (*P* < 0.05). The details are shown in Table 2.

**Table 2:** The univariate analysis of participants' demographic characteristics and mask-wearing behaviors (*n* = 1,972).

**Characteristic**	**Category**	**Different mask-wearing rate [*****n*** **(%)]**				**Scores of mask-wearing behaviors**	***t/F***	***P*** **-value**
		**Ordinary mask**	**Disposable medical masks**	**Medical surgical masks**	**Medical protective masks**			
Gender	Male	207 (42.4)	479 (39.7)	368 (35.3)	228 (43.7)	35.38 ± 5.17	−6.670	0.000
	Female	281 (57.6)	729 (60.3)	675 (64.7)	294 (56.3)	37.08 ± 5.64		
Age	<18	3 (0.6)	8 (0.7)	4 (0.4)	4 (0.8)	29.33 ± 1.44	14.307	0.000
	18–40	345 (70.7)	870 (72.0)	805 (77.2)	371 (71.1)	36.82 ± 5.32		
	41–65	138 (28.3)	327 (27.1)	232 (22.2)	144 (27.6)	35.53 ± 5.93		
	>65	2 (0.4)	3 (0.2)	2 (0.2)	3 (0.6)	33.29 ± 8.34		
Place of residence	City	221 (45.3)	650 (53.8)	530 (50.8)	288 (55.2)	37.79 ± 5.55	79.646	0.000
	Township	118 (24.2)	304 (25.2)	286 (27.4)	119 (22.8)	35.11 ± 5.17		
	Countryside	149 (30.5)	254 (21.0)	227 (21.8)	115 (22.0)	34.43 ± 4.83		
Educational level	Primary school or below	16 (3.3)	69 (5.7)	37 (3.5)	21 (4.0)	33.13 ± 4.55	17.877	0.000
	Junior high school	70 (14.3)	154 (12.7)	113 (10.8)	72 (13.8)	35.03 ± 5.64		
	High school	61 (12.5)	136 (11.3)	125 (12.0)	75 (14.4)	35.43 ± 5.52		
	Undergraduate	259 (53.1)	639 (52.9)	602 (57.7)	281 (53.8)	36.86 ± 5.62		
	Post-graduate (Master/Ph.D.)	82 (16.8)	210 (17.4)	166 (15.9)	73 (14.0)	37.47 ± 4.79		
Marital status	Unmarried	96 (19.7)	400 (33.1)	298 (28.6)	126 (24.1)	39.10 ± 4.55	127.848	0.000
	Married	364 (74.6)	694 (57.5)	667 (64.0)	350 (67.0)	35.35 ± 5.47		
	Divorced	28 (5.7)	114 (9.4)	78 (7.5)	46 (8.8)	33.86 ± 5.59		
Work/living environment	Work in relation to the COVID-19 epidemic^a^	145 (29.7)	364 (30.1)	329 (31.5)	196 (37.5)	37.22 ± 5.56	34.469	0.000
	Work in crowded places^b^	97 (19.6)	356 (29.5)	292 (28.0)	108 (20.7)	37.89 ± 5.57		
	Home quarantine or living with people in self-quarantine	30 (6.1)	46 (3.8)	50 (4.8)	18 (3.4)	36.04 ± 3.46		
	Indoor work/activities/study	78 (16.0)	148 (12.3)	122 (11.7)	62 (11.9)	33.54 ± 4.05		
	Well-ventilated place	56 (11.5)	92 (7.6)	92 (8.8)	60 (11.5)	33.78 ± 5.00		
	Patients in medical institutions	44 (9.0)	108 (8.9)	90 (8.6)	64 (12.3)	37.43 ± 6.44		
	Gather together to study and take activities	38 (7.8)	94 (7.8)	68 (6.5)	14 (2.7)	33.84 ± 3.91		
Flu symptoms	Yes	174 (35.7)	298 (24.7)	266 (25.5)	158 (30.3)	33.15 ± 4.26	−16.272	0.000
	No	314 (64.3)	910 (75.3)	777 (74.5)	364 (69.7)	37.54 ± 5.46		
Living with people in home isolation	Yes	200 (41.0)	322 (26.7)	308 (29.5)	170 (32.6)	33.03 ± 3.78	−18.115	0.000
	No	288 (59.0)	886 (73.3)	735 (70.5)	352 (67.4)	37.72 ± 5.53		
Total		488 (24.7)	1,208 (61.3)	1,043 (52.9)	522 (26.5)			

a *Police, security, courier and other practitioners*;

b *Staff in relatively closed places such as hospitals, airports, railway stations, subways, ground buses, planes, trains, supermarkets, restaurants, etc*.

The mean scores of the participants' mask-wearing behavior was (36.44 ± 5.53) points, with 1,167 showing good behavior and 805 showing poor behavior. The rate of good behavior among the mask-wearing public was 59.2%. Most of participants knew how to wear and use the mask correctly, including identifying the front and back of the mask and the upper and lower sides (74.5%); making sure the mask covers the mouth, nose and chin (75.8%); checking for gaps between the face and the mask (66.4%); never or occasionally hang the mask under the chin (65.3%); never or occasionally expose the nose and mouth to breathe (73.9%); never or occasionally wear multiple masks at the same time (78.5%); and never or occasionally reuse disposable masks (73.1%). However, 41.5% of participants seldom cleaned their hands before wearing a mask, and 52.7% and 59.8% of participants never or occasionally wash their hands after touching or adjusting the mask. The three items with the lowest scores were “Have you adjusted the position of the mask while using it?” (2.31 ± 0.98) points, “Do you wash your hands or use a hand sanitizer immediately after adjusting your mask?” (2.37 ± 0.97) points, and “Do you wash your hands or use a hand sanitizer immediately after touching the mask?” (2.46 ± 0.92) points. The scores for each item are shown in Table 3.

**Table 3:** The characteristics of the public mask-wearing behaviors (*n* = 1,972).

**Items**	**Scores (***$\bar{x}$* ±***s*****)**
Before wearing a mask, can you correctly identify the front and back of the mask and the upper and lower sides?	3.25 ± 1.03
Do you wash your hands or use a quick hand sanitizer before wearing a mask?	2.73 ± 1.06
Do you make sure the mask covers your mouth, nose and chin after you put it on?	3.24 ± 1.01
After wearing the mask, do you check for gaps between your face and the mask?	2.99 ± 1.07
Did you touch the mask while using it?	2.60 ± 0.99
Do you wash your hands or use a quick hand sanitizer immediately after touching the mask?	2.46 ± 0.92
Have you adjusted the position of the mask while using it?	2.31 ± 0.98
Do you wash your hands or use a quick hand sanitizer immediately after adjusting your mask?	2.37 ± 0.97
Do you hang the mask under your chin while using it?	2.72 ± 0.10
Do you expose your nose and mouth to breathe while using the mask?	2.93 ± 0.94
Do you wash your hands or use a quick hand sanitizer right after removing your mask?	2.63 ± 1.01
Will you wear multiple masks at the same time?	3.21 ± 0.94
Do you reuse disposable masks?	3.01 ± 0.94

As shown in Table 4, the e-health literacy scores of the public with good mask-wearing behavior was an average of (29.72 ± 7.22) points, which was significantly higher than those with poor mask-wearing behavior (22.15 ± 5.02, *P* < 0.05). Besides, each item score with good mask-wearing behavior was significantly higher than those with poor behavior (*P* < 0.05). Correlation analysis showed that there was a positive correlation between mask-wearing behavior and e-health literacy (*R* = 0.550, *P* < 0.05).

**Table 4:** The characteristics of public e-health literacy (*n* = 1,972).

**Items**	**Good (*****n*** = **1,167)**	**Poor (*****n*** = **805)**	***t***	***P*** **-value**
**Scores of e-health literacy**	**29.72 ± 7.22**	**22.15 ± 5.02**	**25.738**	**0.000**
**I know how to find useful health resource information online**	**3.73 ± 1.18**	**2.95 ± 1.33**	**13.616**	**0.000**
I know how to use the Internet to answer my own health questions	3.68 ± 1.25	2.73 ± 1.18	17.142	0.000
I know what health resource information is available on the Internet	3.74 ± 1.12	2.62 ± 1.27	20.669	0.000
I know where to find useful health resource information on the Internet	3.81 ± 1.09	2.85 ± 1.24	18.256	0.000
I know how to help myself with the information I get on Internet health resources	3.76 ± 1.14	2.88 ± 1.36	15.708	0.000
I have the ability to evaluate the quality of online health resource information	3.60 ± 1.13	2.72 ± 1.23	16.507	0.000
I can distinguish between high-quality and low-quality health resource information on the Internet	3.72 ± 1.04	2.54 ± 1.25	22.880	0.000
I am confident in using online information to make health-related decisions	3.67 ± 1.07	2.87 ± 1.21	15.387	0.000

Logistic regression analysis was conducted with the behavior of wearing masks as the dependent variable and gender, age, place of residence, educational level, marital status, work or living environment, presence of flu symptoms, and whether living with people in home quarantine as the independent variables. The assignments of dependent variable and independent variables are shown in Table 5. Logistic regression analysis showed that seven factors are related to mask-wearing behavior including gender, place of residence, educational level, marital status, work or living environment, presence of flu symptoms, and whether living with people in home quarantine.

**Table 5:** The assignments of independent variables.

**Variables**	**Assignment description**
Mask-wearing behavior	0 = Good, 1= Poor
Gender	0 = Male, 1= Female
Age	0 = <18, 1= 18–40, 2 = 41–65, 3 = >65
Place of residence	0 = City, 1 = Township, 3 = Countryside
Educational level	0 = Primary school or below, 1 = Junior high school, 2 = High school, 3 = Undergraduate, 4 = Post-graduate (Master/Ph.D.)
Marital status	0 = Unmarried, 1 = Married, 2 = Divorced
Work/living environment	0 = Work in crowded places, 1 = Work in relation to the COVID-19 epidemic, 2 = Home quarantine or living with people in self-quarantine, 3 = Indoor work/activities/study, 4 = Well-ventilated place, 5 = Patients in medical institutions, 6 = Gather together to study and take activities
Flu symptoms	0 = Yes, 1 = No
Living with people in home isolation	0 = Yes, 1 = No

**Figure 2:**
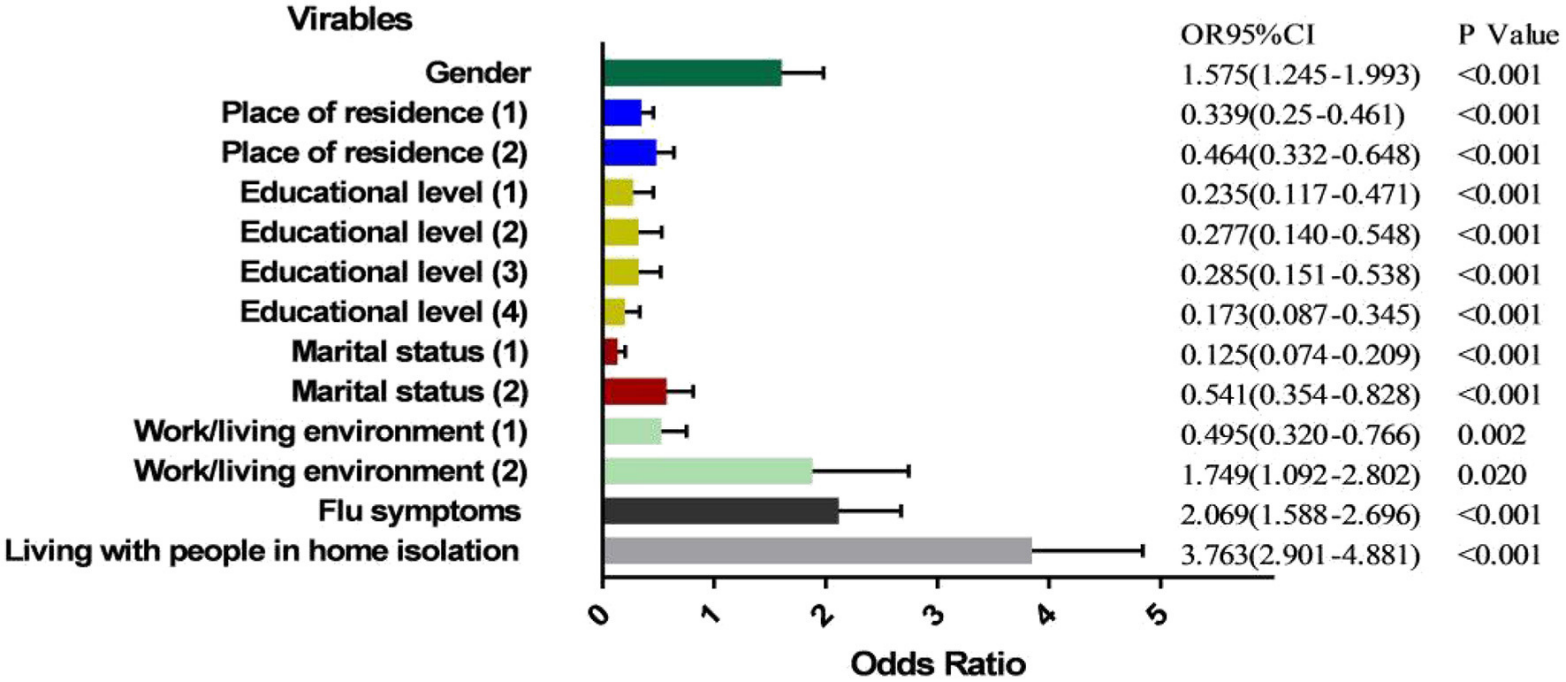
Analysis of influencing factors of the public mask-wearing behaviors. Place of residence (1) = City, Place of residence (2) = Township (Reference category: Countryside); Educational level (1) = Junior high school, Educational level (2) = High school, Educational level (3) = Undergraduate, Educational level (4) = Post-graduate (Reference category: Primary school or below); Marital status (1) = Married, Marital status (2) = Divorced (Reference category: Unmarried); Work/living environment (1) = Patients in medical institutions, Work/living environment (2) = Focus on study or activity (Reference category: Work in relation to the COVID-19 epidemic).

The result of the binary logistic regression was expressed as an odds ratio (OR) with its corresponding 95% confidence intervals (95% CI), and it is a risk factor for mask-wearing behavior which the OR value is >1 and the OR value is <1 is a protective factor for mask-wearing behavior, as shown in Figure 2. The mask-wearing behavior of males were worse (OR = 1.575, 95 CI = 1.245–1.993, *P* = 0.000) compared with female. Compared with countryside, the behavior in cities (OR = 0.339, 95 CI = 0.250–0.461, *P* = 0.000) and townships (OR = 0.464, 95 CI = 0.332–0.648, *P* = 0.000) were better. Compared with those with primary school education and below, those with junior high school (OR = 0.235, 95 CI = 0.117–0.471, *P* = 0.000), high school (OR = 0.277, 95 CI = 0.140–0.548, *P* = 0.000), undergraduate (OR = 0.285, 95 CI = 0.151–0.538, *P* = 0.000), and post-graduate degree and above (OR = 0.173, 95 CI = 0.087–0.345, *P* = 0.000) showed better compliance with wearing masks, while the difference of mask-wearing behavior was not statistically significant between those with junior high school and high school (*P* = 0.444) or between with undergraduate and post-graduate degree (*P* = 0.073). The mask-wearing behavior of unmarried (OR = 0.125, 95 CI = 0.074–0.209, *P* = 0.000) and married (OR = 0.541, 95 CI = 0.354–0.828, *P* = 0.000) people are better than divorced. Compared with the public engaged in COVID-19-related work, the patient's (OR = 0.495, 95 CI = 0.320–0.776, *P* = 0.002) mask-wearing behavior was better, and the public who gathered to study and engage in activities (OR = 1.749, 95 CI = 1.092–2.802, *P* = 0.020) were worse. Besides, the mask-wearing behavior of people with flu symptoms (OR = 2.069, 95 CI = 1.588–2.696, *P* = 0.000) and those living with people in home quarantine (OR = 3.763, 95 CI = 2.901–4.881, *P* = 0.000) were worse than those without these situations. Therefore, male, divorced, gathered to study and engage in activities, flu symptoms and living with people in home quarantine are the risk factor for mask-wearing behavior; living in cities and townships, high education level, patients in medical institutions are the protective factor for mask-wearing behavior.

## Discussion

In this study, it was divided into seven regions to conduct a nationwide sample survey (excluding Hong Kong, Taiwan and Macao) according to the geographical location of China, which covered every province and city. A total of 2,093 participants participated in this survey, and there was no statistical difference in the demographic characteristics of participants in different regions. In the study design stage, we prepared to conduct a sample survey according to the proportion of the population in different regions of China. However, due to the limited number of investigators, this survey was conducted based on the “Wenjuanxing” platform by using convenient sampling methods. After the quality control was carried out, some questionnaires that did not meet the requirements were screened out and excluded, then current sample was obtained.

According to the data of China Statistical Yearbook (22), there is still a certain difference in the population distribution between the sample in this study and the results of China's seventh census. There are more female participants than males in this study, while the actual proportion of males is higher than that of females in China; the proportion of participants under the age of 40, living in the city, undergraduate, post-graduate, unmarried and divorced is higher than that of the real national population-level, while the proportion of participants aged 40 above, living in the countryside, high school and below and married is lower than that of the national population-level. This may be due to the limitations of the sampling method of this study, and due to the influence of the demographic characteristics of the participants themselves, such as male, old age, living in the countryside, and those with lower education level may have lower compliance with questionnaire survey. Besides, since the investigators recruited in this study are mainly concentrated in East China, the participants in East China region are higher than the national population level in this region. Therefore, the above-mentioned bias of the sample may limit the generalization of the results of this study.

Previous studies have shown that during the non-epidemic period in China, the mask-wearing behavior of medical staff and people who have extensive contact with the population is better than that of ordinary residents (23). The results of this study showed that during COVID-19, the proportion of the public wearing masks was 99.8%, of which 59.2% showed good behavior, indicating that the mask-wearing rate of the public during COVID-19 was higher than that during the non-epidemic period. It could be because COVID-19 changed the behavior pattern of mask-wearing residents, and their awareness of travel protection has significantly improved. Besides, it may be related to cultural norms. In China, people have a sense of collectivism; hence, they are seemingly more likely to wear masks in public, as their sense of interdependence may motivate them to safeguard themselves and those around them (24). However, the public's knowledge about the selection, usage, and precautions, and the frequency of changing masks was sufficient but they were less aware of washing hands after touching masks and removing them.

According to “Guidelines for the public rational face mask wearing” (hereinafter “Guidelines”) (19), wearing masks scientifically is an important measure to effectively respond to COVID-19. During COVID-19, the different classifications of the people determine the different risks of environmental exposure and the protection levels of the masks worn are different. The utilization rate of different mask types showed the most used are disposable medical masks (61.3%). Among people engaging in COVID-19-related work such as police, security, courier, and other practitioners, the proportion of those wearing medical surgical masks and medical protective masks was the highest, 31.5 and 37.5%, respectively, while the rest wore more disposable medical masks. This may be related to higher protection awareness and needs under high exposure risks. However, during COVID-19, among people engaging in COVID-19-related work, the proportion of those wearing medical surgical masks and medical protective masks was relatively low. It could be seen that those engaged in COVID-19-related work had a low level of protection awareness. The government, hospitals, and other relevant departments should strengthen the protection of wearing masks and guide people to wear them scientifically to ensure their safety.

For people who worked in crowded places and were often exposed to densely populated places such as airports, railway stations, subways, ground buses, planes, trains, supermarkets, restaurants, “Guidelines” indicated that in medium and low-risk areas, disposable medical or medical surgical masks should be worn, and medical surgical masks or protective masks such as KN95 or N95 should be worn in high-risk areas (19). The results showed that the proportions of people who worked in crowded places choose to wear disposable medical masks when they went out was the highest, 29.5%, followed by medical surgical masks, and medical protective masks, 28.0 and 20.7%, respectively. It showed that there was a gap between the way people choose to wear masks and the requirements for it in the “Guidelines”.

The results of this study showed that during COVID-19, the public had a higher level of e-health literacy, with an overall score of (26.63 ± 7.41) points, and the level of people who wore masks well was significantly higher. The correlation analysis also showed that there was a positive correlation between the mask-wearing behavior and e-health literacy, that is, the higher the level of e-health literacy, the better the mask-wearing behavior. Moreover, this survey showed that all the participants have learned the information on how to use masks, and they preferred to obtain information through social media (WeChat, Weibo, QQ), TV, and the Internet. This could be because people with a high level of e-health literacy pay more attention to their health, and have a higher ability to use the acquired information to solve their health problems. Therefore, it is necessary to support the construction of public health service system infrastructure and increase their e-health literacy skills and knowledge to familiarize them. Moreover, it is important to help the public access and utilize e-health information, and services to improve their e-health literacy level, and enhance self-protection ability.

According to the social-ecological model (25), the public's compliance regarding mask-wearing does relies on social and interpersonal relationships as well as individual characteristics and personal attitudes. During COVID-19, male participants' compliance with mask-wearing was significantly worse than that of female participants. This gender difference may influence the mask-wearing behavior, as men were less likely to believe the virus will seriously affect them and feel weak and ashamed when wearing a mask (26). This study showed that the compliance of people wearing masks in the countryside was worse than that in cities and townships. Fisher et al. (27) showed that wearing masks may not be regarded as important in many countryside areas in the United States during COVID-19 compared to cities. People in the countryside may not even know an individual with the virus and feel that wearing masks are unnecessary. Besides, people with primary school education and below showed poor mask-wearing behavior masks. The reason could be related to the fact that people with higher education have more access to health services, healthcare, and health education, and have a stronger awareness of protection (28).

However, we found that people in different work or living environments showed different mask-wearing behaviors. Patients who went to the hospital for medical treatment have better mask-wearing behaviors. This may be due to concerns about the high risks transmission of COVID-19 when patients seek medical care, thus prompting patients to have better mask-wearing behavior (29, 30). However, the compliance of mask-wearing of those who gathered for study or activities and home quarantine were lower than people engaging in COVID-19-related work. Moreover, when people have flu symptoms, they will experience discomfort such as nasal congestion and labored breathing, thus showing lower mask-wearing compliance. Therefore, it is suggested that the role of mass media and new media in health education and publicity should be brought into full play in the future epidemic prevention and control. Epidemic prevention and control departments, enterprises, institutions, schools, and so on should strengthen popular science education on the public's mask-wearing behavior. It is necessary to increase supervision and punishment in public places such as restaurants, parks, and entertainment venues to prevent and control the development of the pandemic.

## Conclusion

COVID-19 has changed the public's mask-wearing in China and the mask-wearing rate is higher than during the non-epidemic period. The overall compliance of the public's protective behavior in China regarding mask-wearing is generally well and the most used types are disposable medical and medical surgical masks. However, the public's mask-wearing behavior is not ideal, and there are deficiencies in their selection, usage, and precautions. The differences in the public's mask-wearing behavior are related to factors including gender, place of residence, educational level, marital status, work or living environment, presence of flu symptoms, whether living with people in home quarantine, and the higher the level of e-health literacy, the better the mask-wearing behavior. Therefore, it is necessary to strengthen their popularization and education on the prevention and control of COVID-19.

## Data availability statement

The raw data supporting the conclusions of this article will be made available by the authors, without undue reservation.

## Author contributions

WZ and S-FC were responsible for the design of the study, data curation, analysis, and completed to draft the manuscript. K-KL, HL, and H-CS participated in data collection and coordination of the study. X-CZ was responsible for quality control of this study and review of the manuscript. All authors have read and approved the final version of the manuscript.

## Funding

This study was supported by Young and middle-aged scientific research project of Wannan Medical College (WKS2021F03).

## Conflict of interest

The authors declare that the research was conducted in the absence of any commercial or financial relationships that could be construed as a potential conflict of interest.

## Publisher's note

All claims expressed in this article are solely those of the authors and do not necessarily represent those of their affiliated organizations, or those of the publisher, the editors and the reviewers. Any product that may be evaluated in this article, or claim that may be made by its manufacturer, is not guaranteed or endorsed by the publisher.
